# Impact of multiple maize technology package adoption on the production efficiency and food security of smallholder farmers in Ethiopia: Evidence from the Sidama region

**DOI:** 10.1016/j.heliyon.2024.e41280

**Published:** 2024-12-26

**Authors:** Ashenafi Guye, Tewodros Tefera, Million Sileshi, Abdi-Khalil Edriss

**Affiliations:** a Haramaya University, College of Agriculture and Environmental Science, School of Agricultural Economics and Agri-business, Dire Dawa, Ethiopia; b Hawassa University, Daye Campus, Department of Agribusiness and Value Chain Management, Daye, Ethiopia; c REFOOTURE Ethiopia Project Manager and Co-strategic Lead, Addis Ababa, Ethiopia; d Lilongwe University of Agriculture and Natural Resources, Department of Agricultural Economics, and African Center of Excellence, Agricultural Policy Analysis–Big Data, Lilongwe, Malawi

**Keywords:** Improved seed, Chemical fertilizer, Row planting, Adoption impact, Production efficiency, Food security, MESR model

## Abstract

This study aimed to investigate the individual or combined impacts of multiple maize technology package adoption on the production efficiency and food security of smallholder farmers in the selected districts of Sidama region of Ethiopia. The cross-sectional data of 424 sample farmers owing 545 maize plots were collected using multistage sampling approaches. The selection-bias corrected multinomial endogenous switching regression (MESR) model was employed to assess the impact of improved maize seed, chemical fertilizers, and row planting adoption on the production efficiency and food security of smallholder maize-producing farmers. In the first stage of the MESR model, the multinomial logit was used to examine the determinants of adoption. The results of the model showed that a male household head, greater household size, land size, tropical livestock unit, oxen, several plots, extension contact, credit access, and membership in farmers-based organizations were significant factors affecting the adoption of improved maize seed, chemical fertilizer, and row planting. In addition, the results from the second stage impact estimation demonstrated that the adoption of maize technology packages, both individually and in combination, significantly impacts the production efficiency and food security of farming households. Moreover, the simultaneous adoption of two or more technology packages has a greater impact than it does in isolation. The highest technical, allocative, and economic efficiency and food consumption scores, which are 33 %, 37 %, 47 %, and 17, respectively, were achieved when farm households adopted all technology packages simultaneously, followed by the adopters of two packages in combination. Therefore, the policies and strategies aimed at increasing farmers’ production efficiency and food security should address the important variables identified and promote wider adoption in combination to realize the utmost benefit.

## 1 Introduction

Agriculture rests among the main powers accelerating the economic progress of Ethiopia. Ethiopia has been experiencing the fastest economic growth in Africa, although it is among the poorest countries of the world [1]. Agriculture contributes a huge portion to the performance of the national economy. At present, Ethiopian agriculture offers about 32.4 percent of the gross domestic product (GDP), 75 percent of foreign currency, 73 percent of employment, and 70 percent of unprocessed products for domestic industries [2,3]. Thus, Ethiopian economic progress strongly depends on the success of the agricultural sector, as it is the basis of the economic growth of the country [4,5]. On the other hand, the performance of the sector has remained below the expected level, and low-level productivity is one of its key features [6]. The potential reason behind low productivity may be market disintegration, scarce wealth, suboptimal use of resources, low adoption, inadequate subsidies, low soil fertility, subsistence farming, exposure to unexpected shocks, and dependence on old-fashioned and rainfall-based farming, among other factors [4,[7], [8], [9], [10]]. These constraints, coupled with sporadic political instability, civil war, and the severe rainfall shortages [1], have aggravated the challenges and kept the sector's potential crop productivity minimal [11].

Moreover, agriculture is subjugated by the farmers' who owned smaller hectares of land and cultivates for home consumption and suffer from chronic poverty and hunger [7]. The agricultural system has not been adequately designed in a way that meets the demand of the rapidly growing and urbanizing population for food and to uphold pervasive economic growth [8]. Despite the efforts of the government and development partners to improve the agricultural performance, the country remains unable to meet the food requests of its people [12]. As a result, ensuring food security and eradicating poverty remain main development agendas in Ethiopia. On the other hand, maintaining food security and poverty reduction are still unthinkable without advanced agriculture production systems [6]. In this regard, the optimal use of available production inputs and technology is supposed to be one of the best strategies for enhancing the efficacy of farmers’ production on small land sizes [13]. Thus, the efficient use of available resources and technologies has received the paramount focus [14]. In addition, yield-enhancing agricultural technologies are important tools for improving farm productivity and reducing food insecurity conditions of small-scale farmers [15]. Consequently, the adoption of an agricultural technology package was expected to offer a substantial portion in improving agricultural growth and the welfare of farm households through increased agricultural output [[16], [17], [18], [19], [20]]. Nevertheless, the agricultural (maize) technology adoption remains at a low level in Ethiopia, and this is also true in the current study sites like Sidama. For example, according to Ref. [21] the improved maize seeds, chemical fertilizers, and row planting adoption rate were 54 %, 45 %, and 44 %, respectively, in the study area. This shows that the evidence on the determinants of agricultural technology adoption in maize production and its impact on the production efficiency and food security status of farm households is still lacking. Thus, policies and strategies based on empirical evidence are important to encourage the adoption of the technology packages and to realize the adoption impact in a broader range. Some recent studies have examined the impact of agricultural technology adoption in maize production and emphasized only one component of the technology package [11,17,18,[22], [23], [24], [25], [26], [27], [28], [29], [30]]. However, assessing the impacts of a single component of the technology package (e.g., maize varieties) misses the significance of other technology packages that may have correlations and under/overestimates the adoption impact. In practice, farmers adopt two or more technology packages simultaneously. Nevertheless, the adoption impact of multiple (two or more) agricultural (maize) technologies has rarely been investigated. In addition, the previous studies investigated on the adoption impact emphasized income, consumption expenditure, maize yield, and others as outcome variables. However, the adoption impact on production efficiency does not exist. If there exists, it is so rare and uncovers maize belt areas like Sidama, which is the current study site.

In terms of econometric techniques, many of the previous studies adopted ordinary least squares (OLS) or propensity score matching (PSM) methods to assess the impact of adoption and failed to consider self-selection bias and endogeneity problems due to specification problem [17,28,29,31]. This study, however, adopted a selection bias-corrected MESR model to assess the impact of multiple maize technology package adoption on the production efficiency and food security of smallholder farmers. Maize (*Zea mays* L.) is one of the strategic staple cereal crops cultivated on 2.5 million hectares by over 9 million smallholders, harvesting about 10.3 million metric tons in 2021 [32]. Approximately 88 % of their production is consumed at home, and maize consumption provides about 29 % of energy content from overall cereals [33,34]. In terms of hectares of land allocated, maize positioned second and first in quantity produced. The national average yield of maize was 42 q/ha in 2021 [32], which is still lower than 56.6 q/ha on average recorded at the world level, even though there has been progress as compared to the average yield over the last four years, which is 39 q/ha [35]. This indicates that the country has been producing yields under the possible achievable output. Thus, the strategies to improve maize productivity are adopting maize technology packages and reducing the inefficiency of resource use, among others. Therefore, this study aimed to assess the impact of multiple maize technology package adoption on the production efficiency and food security of maize-producing households in the northern Sidama zone of Ethiopia. The impact analysis offers critical insights for governments, policymakers, and development partners when organizing and developing effective adoption-promotion strategies for poverty reduction and food security.

## 2 Research approaches

### 2.1 Description of study areas

**Fig. 1:**
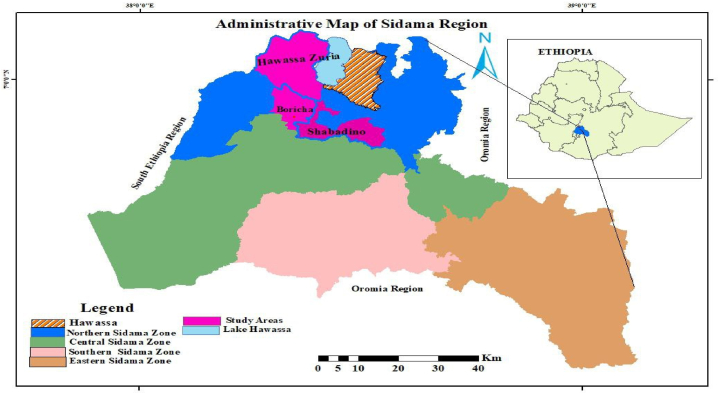
Study area map.

Three woreda^1^ were randomly chosen from the potential maize-producing woredas of the Sidama region. There are 12 regions in Ethiopia, including Sidama region. The Sidama region is situated in the Southeastern part of the country, 275 km from the capital. It borders National Regional State of Oromia to the north, northeast and southeast and Central Ethiopia region in the south and west. Sidama is a region with a high population density of 674 person/km.^2^ The livelihood of rural communities’ rests on small-scale crop production and livestock keeping. The Sidama region is categorized among regions recognized for their maize production potential [36]. However, the agricultural innovations uptake in maize production remains at a low level [21]. Fig. 1 depicts the geographical situation map of the current study site.

### 2.2 Data sources, types, and collection techniques

The necessary data for this study were collected from primary (firsthand) as well as secondary sources. The firsthand data source comprises the demographic information of sample respondents, socioeconomic factors, institutional setting, and infrastructural aspects, maize plot-level traits, and many other issues related to agricultural innovations’ adoption. The primary data was gathered via a semi-structured questionnaire. The questionnaire was pretested on twelve randomly selected sample households, and the necessary changes were performed to proceed with official data collection. The researcher trained, recruited, and supervised data collectors who were familiar with the research sites, spoke the local language, and had prior data collection expertise. Secondary source data were obtained from the Central Statistical Agency, the Sidama Regional Bureau of Agriculture, and the Plan and Development Commission Bureau. Other secondary materials were gathered from published and unpublished sources.

### 2.3 Sampling approach

Multi-stage sampling approaches were followed to select the representative sample from the total maize-producing farmers of the study areas. First, Hawassa Zuria, Boricha, and Shabadino woredas were randomly chosen from woredas known for maize production potential in the Sidama region. Next, 5 kebeles^2^ from Hawassa Zuria, 2 kebeles from Boricha, and 4 kebeles from Shabadino woreda were randomly selected using the lottery method based on the number of kebeles each woreda contains (Table 1). Lastly, a total of 424 respondents were selected randomly from the maize producers’ sampling frame based on the PPS^3^ of each kebele. In addition, plot-level data were collected from the sample respondents, resulting in 545 plots.

**Table 1:** Frequency of maize-producing farmers’ across kebele and woreda.

Districts	Kebele/Village	Maize producers	Sample farmers
Hawassa Zuria	Jara qarara	953	63
	Jara damuwa	337	22
	Labu koromo	890	59
	Doyo otilicho	842	56
	Sama ejersa	523	35
Boricha	Qonsore chafa	554	37
	Qonsore goge	510	34
Shabadino	Sadeqa	608	40
	Alawo ano	378	25
	Qonsore ano	454	30
	Morocho shondolo	348	23
	Total	6398	424

### 2.4 Data analysis techniques

#### 2.4.1 Stochastic production function (SPF)

To estimate the technical efficiency of the maize-producing households, the SPF was adopted following, Battese and Coelli [37] as specified in equation (1):

(1) $$\begin{array}{cc} \ln\;Y_{i}=f\left(X_{i}\beta \right)+\left(\nu_{i}-\mu_{i}\right) & i=1,2,\ldots .,N \end{array}$$

The technical efficiency of farm households (Teffi) could be computed as the percentage of observed output ($Y_{i}$) to maximum output ($Y_{i}^{*}$) as shown in equation (2):

(2) $${Teff}_{i}=\frac{Y_{i}}{\exp\;\left(X_{i}\beta +\nu_{i}\right)}=\exp\left(-\mu_{i}\right)=\frac{Y_{i}}{{Y_{i}}^{*}}\;;\;Where\;0\leq {Teff}_{i}\leq 1$$

The empirical Cobb − Douglas production function adopted in this research is given as equation (3):

(3) $$\ln\left(Y_{i}\right)=\beta_{0}+\beta_{1}\text{lnLand}+\beta_{2}\text{lnFertilizer}+\beta_{3}\;\ln\;Labor+\beta_{4}\;\ln\;Seed+\beta_{5}\;\ln\;Oxen+{\;\beta }_{6}\;\ln\;agro-chemicals+\left(\nu_{i}-\mu_{i}\right)$$

Where $\ln\;{\;Y}_{i}$ represent actual maize output, land (ha), labor (ME/days), fertilizer (kg), seeds (kg), oxen (number), and agrochemicals (liter) are production inputs; $\beta_{0}$ is a constant; $\beta_{i}$ represents the coefficients assessed; and $\nu_{i}\;\&\;\mu_{i}$ represents the noise term causing inefficiency.

#### 2.4.2 The stochastic cost function (SCF)

To assess the economic efficiency of the farm households in the study area, the stochastic cost frontier was specified following [38], as specified in equation (4):

(4) $${CST}_{i}=f\left(Z_{i},Y_{i};\alpha \right)\exp\;\left(\delta +\mu \right)$$

The economic efficiency (Eeffi) could be computed as the ratio of the observed cost (${OC}_{i}$) to the maximum cost (${FC}_{i}^{*}$), as given in equation (5).

(5) $${Eeff}_{i}=\frac{{AC}_{i}}{{FC}_{i}^{*}}\;;where\;0\leq Eeffi\leq 1$$

The empirical Cobb − Douglas stochastic cost function used in this study can be defined as equation (6):

(6) $$\begin{array}{cc} {lnC}_{i}=\alpha_{0}+\alpha_{1}\;\ln\text{CL}+\alpha_{2}\;\ln\text{CS}+\alpha_{3}\;\ln\text{CF}+\alpha_{4}\;\ln\;\text{CLa}+\alpha_{5}\;\ln\text{CO}+\alpha_{6}\;\ln\text{CA}+ & \alpha_{7}\;\ln\;\left(Y_{i}\right)+\left(\nu_{i}+\mu_{i}\right) \end{array}$$

Where, ${lnC}_{i}$ is the least cost incurred for producing maize; $Y_{i}$ is the total maize output in kg; lnCL−lnCA represents the cost of land (rental value), seed, labor, fertilizer, oxen and agro-chemicals; and $\alpha^{\prime }s$ represents the parameters to be estimated. Moreover, following the specification defined by Ref. [38], this study estimated the allocative efficiency (Aeffi) as stated in equation (7):

(7) $$A{eff}_{i}=\frac{E{eff}_{i}}{T{eff}_{i}}\;;Where\;0\leq Aeffi\leq 1$$

#### 2.4.3 Food consumption score (FCS)

FCS was used to estimate the food security condition of the farm households in the study area. FCS is a composite variable that is composed of food consumption rate, dietary variety, and the relative nutritive value of each food item [39]. Relevant information on food items that farm households consumed within a one-week reference period or seven days before the data gathering period were collected. The FCS was then estimated by interviewing the sample respondents about how many times they consumed from 8 food bundles within the recall duration before the data collection point of time. The frequency of consumption of each food item was added and computed based on the value assigned to each food group. Finally, the sample maize-producing households were classified into one of three categories based on the FCS score they attained, and threshold points developed by WFP [39,40]. These are poor food consumption scores ranging from 0 to 21, borderline food consumption scores, which also range from 21.5 to 35, and acceptable food consumption score, which is ≥ 35.

#### 2.4.4 The multinomial endogenous switching regression (MESR) model

The impacts of improved seed, chemical fertilizers, and row planting adoption on farmers' production efficiency and food security were estimated adopting the MESR model following recent studies [[41], [42], [43], [44], [45], [46], [47]]. Impact analysis based on nonexperimental data is challenging due to selection bias. However, the previous studies [17,28,29,31] adopted ordinary least squares (OLS) or propensity score matching (PSM) to assess the adoption impact and missed considering both observed and unobserved factors. Hence, these methods were not adequate for estimating the true impact of adoption [9,22,27,48]. In a situation where selection bias is a problem and more than two categories exist the MESR model is appropriate [48,49]. In this model, the analysis was undertaken in a two-stage process. In the first stage of the impact model, the multinomial logit was used to identify the determinants of adoption. In addition, the inverse Mills ratio (IMR) was generated in this stage as additional covariates to account for unobserved heterogeneity. The farmers’ utility that is obtained from the adoption of the maize technology is a function of many independent factors and error terms. However, the utility derived from the adoption is unobserved, but only its choice of the technology package is observed. Let us consider the following latent variable to construct a theoretical utility function, as given in equation (8).

(8) $$A_{ij}=Z_{i}\beta_{J}+\varepsilon_{ij}$$

Where $A_{ij}$ is the unobserved variable that denotes the $i^{th}$ farmer's utility attained by adopting technology j (j = 1, 2, … …, m) given alternative M. $Z^{\prime }s$ are groups of explanatory variables, and $\varepsilon_{ij}$ are unobserved factors. The farm household's choice of any of the maize technology package is represented by $A_{ij}$ and specified in equation (9) as follows:

**Figure:**
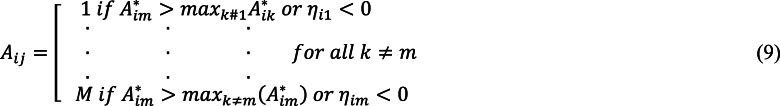

The $i^{th}$ farm household will select the technology packages j given the package k if the utility obtained from technology package ($A_{j})$ is higher than the utility derived from the older one ($A_{o}$), i.e., $A_{ij}=A_{j}-A_{o}>0$. The farmers’ choice probability of technology package j was computed using a multinomial logit model (MNL) $P_{ij}$, following the works of [41,43,[45], [46], [47]] and specified in equation (10) as follows:

(10) $$P_{ij}=P\left(\varepsilon_{ij}\left\langle 0\right|X_{i}\right)=\frac{\exp\left(X_{i}\alpha_{k}\right)}{\sum_{k}^{m}\exp\left(X_{i}\alpha_{k}\right)}$$

Where j = 1, 2, ….M.

Furthermore, in the second stage of the MESR model, the impact of adoption on the production efficiency and food security was estimated using ordinary least squares. Four outcome equations were estimated with and without adoption. The relations between production efficiency and food security indicators ($Y_{i}$) and the explanatory variables ($X_{i}$) were examined for the selected maize technology packages. The outcome variable ($Y_{i}$) is observed if and only if one of the technology j has been adopted [50,51]. In this study, there are eight adoption categories, and one of the categories was assigned as a base category (j = 0). Non adoption was a reference category in this investigation. For the other combinations (j = 1, 2…, M), minimum of one technology package is adopted. To assess the impact of adoption on outcome variables for each combination of the j strategy, the m equations were given as equations (11a) and (11b):

**Figure:**
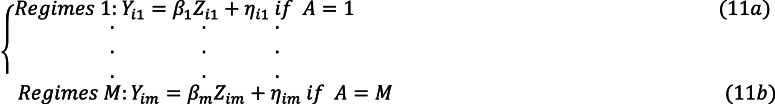

Where $Y_{i}$ represents Teffi, Aeffi, Eeffi, and FCS of the $i^{th}$ farmer in the regime; Z represents explanatory variables; and $\eta_{im}$ represents the error terms, $N∼\left(0,\sigma_{m}^{2}\right)$. If the two error terms ($\varepsilon_{ij}\;and\;\eta_{ij}$) are correlated $\left(E\;\left(\varepsilon_{ij}\eta_{ij}\neq 0\right)\right)$ and not normally distributed with zero mean and constant variance, ($N\neq (0\text{,}\sigma_{m}^{2}$), it is important to include the selection correction terms (λ) in each of the technology package choices to obtain consistent OLS estimates of the parameters. Following [51], the selectivity bias correction term or inverse Mills ratio (IMR), could be estimated from equation (10) and given in equation (12) as follows:

(12) $$\lambda_{im}=\sum_{k\neq m}^{j}\rho_{m}\left[\frac{{\hat{P}}_{ki}\;\ln\left({\hat{P}}_{ki}\right)}{1-{\hat{P}}_{ki}}+\ln\;{\hat{P}}_{mi}\right]$$

Where ρ (rho) is the correlation between $\varepsilon_{im}$ and $\eta_{im}$. To account for selection bias, the selectivity term (λ) was incorporated into equations (11a) and (11b) following [48] and specified as follows in equations (13a) and (13b)

**Figure:**
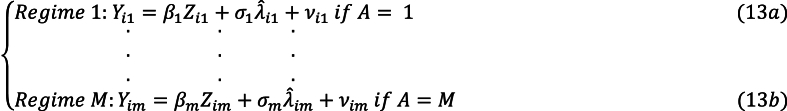

Where σ is the covariance between $,\varepsilon_{im}$*,* ${\;\eta }_{im}$, and $\nu_{im}$ is the disturbance term with an anticipated value of 0; ${\hat{\lambda }}_{1i}$ & ${\hat{\lambda }}_{2i}$ are IMR estimated from the first stage estimation. The standard errors in equations (11a) and (11b) were bootstrapped to consider the heterogeneity of variance that arises from (λ) regressor generation in a two-stage estimation process. Besides, the variables in the first stage of impact model must consist instrumental variable(s) for equation (10) to be identified and to improve the robustness of the identification [48,52]. Thus, extension contact, main market center distance, maize plots distance, and farmers' training center distance were included as instrumental variables. Admissibility test was conducted to prove the validity of instruments variables [53] and they found to be valid selection instruments. This indicates that they influence the adoption decisions but do not influence the production efficiency and food security indicators of non-adopters directly. The plausible reason is that these variables were found to influence decision to adopt or not to adopt significantly [Model 1: Wald chi2 (28) = 117.25 (Prob>chi2=0.0000)]. However, they do not have direct relationship with production efficiency and food security indicators of non-adopters, otherwise via adoption decision [Model 2: F-stat^1^ = 0.70 (Prob > F = 0.5968), F-stat^2^ = 0.26(Prob > F = 0.9058), F-stat^3^ = 1.57(Prob > F = 0.1854), F-stat^4^ = 0.55 (Prob > F = 0.6992)]. Recent studies by Refs. [41,44,[53], [54], [55]] have used one or more similar variables in impact estimation as instruments.The conditional expectations for production efficiency and food security can be computed as follows:

Farm households with the adoption of the maize technologies are given as equation (14a) (real)^4^

(14a) $$E\left(Y_{1i}|A_{i}=1\right)=X_{1i}\beta_{1}+\sigma_{1\eta }\lambda_{1i}$$

Farm households without adoption of the maize technology are specified as equation (14b) (real):

(14b) $$E\left(Y_{2i}|A_{i}=0\right)=X_{2i}\beta_{2}+\sigma_{2\eta }\lambda_{2i}$$

Farmers that did adopt the maize technology had decided not to adopt can be expressed as equation (14c) (not real):

(14c) $$E\left(Y_{2i}|A_{i}=1\right)=X_{1i}\beta_{2}+\sigma_{2\eta }\lambda_{1i}$$

Farmers that did not adopt any of the maize technologies decided to adopt can be given as equation (14d) (not real):

(14d) $$E\left(Y_{1i}|A_{i}=0\right)=X_{2i}\beta_{1}+\sigma_{1\eta }\lambda_{2i}$$

Following Ref. [56] the average treatment effect on the treated (ATT) was computed as difference between equations (14a), (14c)) as stated in equation (15).

(15) $$ATT=E\left(Y_{1i}|A_{i}=1\right)-E\left(Y_{2i}|A_{i}=1\right)$$

$$=X_{1i}\left(\beta_{1}-\beta_{2}\right)+\left(\sigma_{1\eta }-\sigma_{2\eta }\right)\lambda_{1i}$$

Likewise, the average treatment effect on the untreated (ATU) was computed as the difference between (14d) and (14b) as specified in equation (16).

(16) $$ATU=E\left(Y_{1i}|A=0\right)-E\left(Y_{2i}|A_{i}=0\right)$$

$$=X_{2i}\left(\beta_{1}-\beta_{2}\right)+\left(\sigma_{1\eta }-\sigma_{2\eta }\right)\lambda_{2i}$$

The most efficient “semi-log”stata command was used for assessing the adoption impact on farmers' technical, allocative, and economic efficiency and food consumption scores of maize-producing farmers [43,45,51]. The smallholders' maize technology package adoption status and its impact on the aforementioned outcome variables are discussed in the following section.

## 3 Results and discussion

### 3.1 Multiple maize technology package adoption status in the study area

**Fig. 2:**
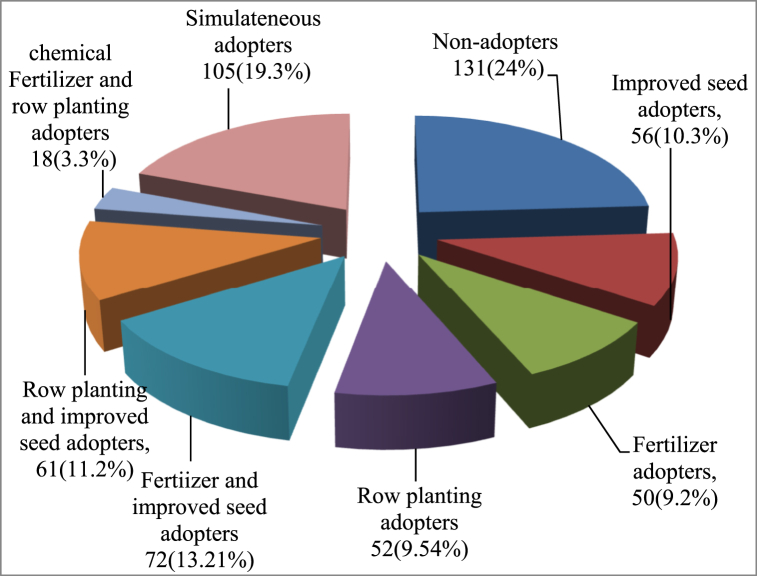
Adoption rate of maize technology packages.

In this study, adopters can be defined as farmers that adopted at least one of the maize technology packages in any of the maize plots. The maize technologies included in this study were improved seed, fertilizers, and row planting, providing eight (2^3^) possible combinations (Fig. 2). Among the 545 maize plots, about 24% were nonadopters. However, improved seed only, chemical fertilizer only, and row planting only were adopted for approximately 10.24 %, 9.17 %, and 9.54 % of the maize plots, respectively. Joint adoption of improved seed and chemical fertilizer only, improved seed and row planting only, and fertilizer and row planting only were adopted for about 13.21 %, 11.19 %, and 3.30 % of the maize plots, respectively. All technology packages were concurrently practiced on 19.27 % of the maize plots. These results showed the highest proportion of packages with the adoption of full technology sets and the combined adoption of improved maize seed and chemical fertilizer only from the adopter category, suggesting that the benefits expected by farmers from these combined technology packages may be greater than those of the others.

### 3.2 Potential challenges of maize technology package adoption

**Fig. 3:**
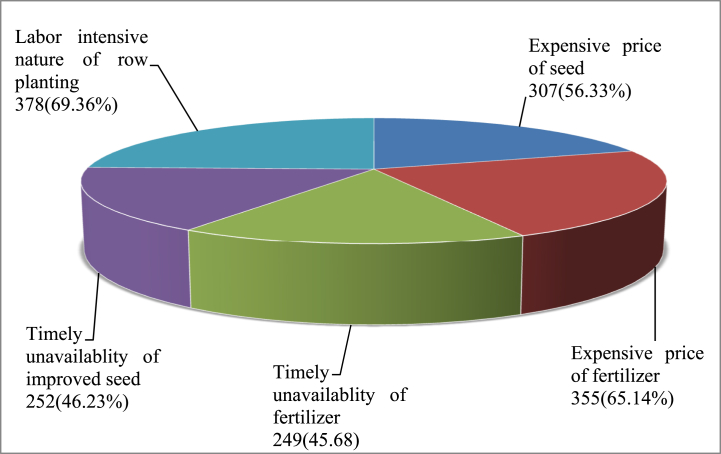
Potential challenges of adoption in the study area.

This study endeavored to discover the potential challenges of adoption in the study areas. Farm households have reported that the steeply rising price of fertilizer and improved seed, the unavailability of the production inputs on time, and the labor-intensive nature of row planting were the main challenges that limited their interest in adoption. As shown in Fig. 3, out of the total sample households, 65.14 % and 56.33 % reported that the expensive price of fertilizer and improved maize seeds, respectively, were barriers to them not adopting the packages. Similarly, 46.23 % and 45.68 % of the respondent farmers have reported the unavailability of improved maize seed and chemical fertilizer on time, respectively, were another challenges that limited their adoption level. Moreover, 69.36 % of the total sample households reported that the labor-intensive nature of row planting has delayed its adoption in the study area. Fig. 3 below portrays the potential challenges of adoption in the study areas.

### 3.3 Summary statistics of the outcome variables

The summary statistics of the outcome variables used in the impact assessment are reported in Table 2. The maize-producing farmers achieved an average technical efficiency (Teffi) of 63.8 %, with a minimum of 13.7 % and a maximum of 94.7 %. This showed that farmers could reduce inputs (land, oxen, seeds, and fertilizers) by an average of 36 percent if they were technically efficient. Similarly, the average allocative efficiency (Aeffi) of sampled farm households was 74 %, ranging from 19.7% to 99.94 %. The allocative efficiency of farm households implies that with available technology and a resource base, it is possible to increase Aeffi by 26 %. In another form of analysis, the mean allocative efficiency of 74 % means that the current level of allocative efficiency could be improved. For example, farm households with an average level of Aeffi would realize a cost minimizing by 26 % derived from (1–0.74)∗100, which helps them to achieve the maximum level of cost efficiency. The pooled outcome of Teffi and Aeffi showed that the average economic efficiency (Eeffi) level was 58.7 %. These results imply that the farm households with the current level of average economic efficiency (51 %) could save approximately 41% of the cost that is derived from (1-0.59)∗100 to attain the maximum level of economic efficiency. Therefore, the findings revealed the presence of considerable technical, allocative, and economic inefficiency of maize-producing farmers. Moreover, the sample farming households obtained a food consumption score of 33.3 on average, suggesting that the farmers attained the score below acceptable food consumption level and they were food insecure.

**Table 2:** Descriptive statistics of the outcome variables.

Outcome variables	Average	Standard deviation	Minimum	Maximum
Technical efficiency(Teffi)	0.6381	0.1577	0.1369	0.9471
Allocative efficiency(Aeffi)	0.7403	0.1967	0.1916	0.9994
Economic efficiency(Eeffi)	0.5868	0.2029	0.1781	0.9186
Food consumption score(FCS)	33.295	9.7586	14	75.5

### 3.4 Summary statistics of the FCS category of farm households

This study revealed that 41.7 % of sample respondents had acceptable (≥ 35) FCSs of 42.7 on average, which indicates that about 42 of the sample farmers were relatively food secured. Whereas, 12.8 % of sample respondents had poor (0–21) food consumption scores of 20.32 on average, which further suggests that about 13 % of the smallholders confront with food insecurity. Likewise, 45.5 % of the sample households had borderline (21.5–35) FCS, which is 28.35; this also showed that approximately 46 % of the maize-producing farmers fall under food insecurity position (Table 3). In general, these research results informed that over 58 % of sample farmers were food insecure.

**Table 3:** FCS categories of the sample farmers.

FSC category	Freq. (%)	Mean	SD	Min	Max
Poor FCS (0 − 21)	12.84	20.32857	1.512953	14	21
Borderline FCS (21.5 − 35)	45.50	28.35685	3.741207	21.5	34.5
Acceptable FCS (≥ 35.5)	41.65	42.68282	6.760656	35.5	75.5

### 3.5 Teffi, Aeffi, and Eeffi and FCS of smallholder farmers by adoption categories

**Fig. 4:**
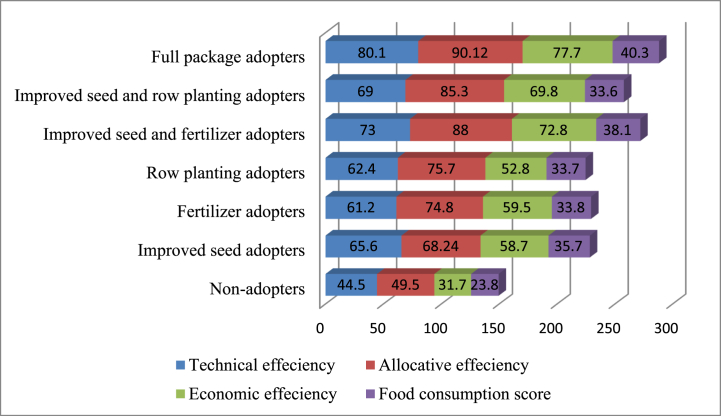
Teffi, Aeffi, and Eeffi and FCS by adoption categories.

As showed in Fig. 4, the study estimated the Teffi, Aeffi, and Eeffi and FCS across adoption categories of maize technology packages. The highest average technical efficiency of 80 % was obtained by adopters of the full technology package, followed by an average technical efficiency of 73 % obtained by farm households that did adopt a combination of improved maize seed and chemical fertilizer. However, the lowest technical efficiency was scored with the adoption of chemical fertilizer in isolation, which was 61.2 %, and non-adopters had average technical efficiency of 44.5 % on average. This implies that there is still substantial technical inefficiency among categories that can be increased with the existing inputs and technologies.

Similarly, full package adopters achieved the highest average allocative efficiency of 90.12 %, followed by an average Aeffi of 88 % obtained by farm households that adopted both improved maize seed and chemical fertilizer. The lowest average allocative efficiency was attained with the adoption of improved maize seeds, which were 68.24 %, and non-adopters with Aeffi of 49.5 %. The results imply that there is still the possibility to increase the present levels of allocative efficiency among adoption categories. In addition, the highest average economic efficiency of 77.7 % was also obtained by full technology package adopters followed by the average economic efficiency of 72.8 % achieved by the adopters of improved maize seeds and chemical fertilizers in combination. However, the lowest average Eeffi of 52.8 % and 31.7 % were obtained by the adopters of row planting only and non-adopters, respectively. These findings indicate that farmers with an average level of Eeffi could minimize the cost by 22.3–68.3 % to attain the maximum level of economic efficiency.

Concerning food consumption score (FCS), adopters of full technology packages obtained an average FCS of 40.3, followed by farm households that did adopt the combinations of improved maize seed and chemical fertilizer, whose FCS is 38.1. However, the lowest FCS is obtained by farmers that adopted improved maize seed and row planting in combination, which were 33.6, and non-adopters FCS, which were 23.8. In general, these results demonstrate that smallholder farmers should adopt maize technologies in combination rather than in isolation to improve production efficiency and food security.

### 3.6 Summary statistics of the dependent variables included in the model

Table 4 reports the descriptive statistics of the explanatory variables included in the analysis. The choice of these variables was depend on important literature review [11,17,18,[22], [23], [24],[26], [27], [28]]. In this study, approximately 95 % of the respondent farmers were male-headed, and their mean age was approximately 45, ranging from 24 − 84 years. The mean years of formal education attained by the farm household were 5.1 years, even though about 24 % of the sample households were illiterate. Sample farmers have a family size of 4.72 adult equivalents on average, ranging from 1.75− 9.75AEs. Plot size cultivated by maize farmers was 0.46 ha on average. Likewise, the sample respondent owned 1.28 maize plots on average. The respondent households owned tropical livestock units of 2.85TLUs on average, with a maximum of 15.75TLUs. In this study, the sample farmers earned ETB^5^10099.68 on average from off-farm activities. Farm households visited extension office 2 times per maize production season on average, with a maximum of 8 times. Moreover, approximately 53 % of sample farmers had credit access, and 71 percent of the total respondents were members of farmer-based organizations. The sample farm households in the study area walked for 20 minutes on average to access the main road, 51 minutes on average to access the main market center, 15 minutes on average to reach the FTC, and 5.28 minutes on average to reach maize plots. The descriptive statistics of explanatory variables are described in Table 4.

**Table 4:** Summary statistics of the explanatory variables included in the analysis.

Explanatory variables	Mean	SD	Min	Max
Sex of the household head (1 = male)	0.9449	0.2282	0	1
Education level (grade)	5.0715	4.1110	0	15
Age of the household head (years)	45.766	11.382	24	80
Family size (in adult equivalents)	4.7235	1.5164	1.75	9.65
Total livestock unit owned (TLUs)	2.8582	2.5976	0	15.75
Ox(en) (number)	0.8678	0.7888	0	3
Off-farm employment(ETB)	10099.68	20175.62	0	160000
Credit access(1 = yes)	0.5302	0.4995	0	1
Extension visit (frequency)	2.0935	2.2361	0	8
Membership in institutions (1 = member)	0.7137	0.4524	0	1
Distance to the main road (in minutes)	19.928	23.589	1	90
Distance to a market center (in minutes)	50.680	20.313	4	105
Distance to FTC (in minutes)	14.846	11.440	1	90
Plot number (number)	1.2844	0.4515	1	2
Plot distance (in minutes)	5.2830	12.225	1	120
Plot size (in hectares)	0.4626	0.4016	0.125	5

### 3.7 Econometric estimations

#### 3.7.1 Determinants of individual or combinations adoption of maize technologies

The first stage of MESR results on the factors affecting improved seed, chemical fertilizer, and row planting adoption individually or in combination were reported in Table 5. Non adoption is a base category (IS_0_F_0_R_0_), where the results of alternative maize technology package adoption are in comparison. The model fits the data reasonably well, with the Wald test chi2 (96) = 2890.21, p = 0.000), the assumption that all the coefficients are jointly equal to zero is rejected. The model outputs revealed that the sex of the household head related positively and significantly with row planting adoption (IS_0_F_0_R_1_). This is probably because row spacing is a labor-intensive technology compared with other maize technologies, and farmers in the study area prepare row spacing manually. Thus, male-headed households have better physical strength and social networks to cope with labor shortages. This result is consistent with the results of [57]. Family size is also linked positively with chemical fertilizer adoption (IS_0_F_1_R_0_). Family size is supposed to be the source of labor accessibility, and farmers with greater family members solve labor shortages and are more likely to adopt agricultural innovations than fewer family size. This finding is in agreement with the results reported by Refs. [25,58]. Farm households with greater livestock units (TLUs) adopted a combination of improved maize seed and chemical fertilizer (IS_1_F_1_R_0_), improved maize seed and row spacing (IS_1_F_0_R_1_), and a full technology package (IS_1_F_1_R_1_) than their counterparts did. In rural parts of Ethiopia, farmers who owned bigger livestock size are considered as wealthy and gain higher social status. This helps them to access inputs and information, as well as better networked. In addition, farm households used part of the income from livestock sales for commercial input purchases and better practice the innovations. The result is consistent with previous findings reported by [26,57]. In addition, oxen ownership was positively and significantly related with the adoption of improved seeds only (IS_1_F_0_R_0_), and to a combination of improved seeds and fertilizer, improved seed and row planting, and a full technology package. Ox (en) ownership concurrently solves the problems of labor and money constraints. The result is consistent with the report of [59].

**Table 5:** First stage MESR model estimation results of the factors affecting multiple maize technology adoption.

Variables	IS_1_F_0_R_0_	IS_0_F_1_R_0_	IS_0_F_0_R_1_	IS_1_F_1_R_0_	IS_1_F_0_R_1_	IS_1_F_1_R_1_
	Coefficient(SE)	Coefficient (SE)	Coefficient(SE)	Coefficient (SE)	Coefficient (SE)	Coefficient (SE)
Sex_hh	−0.0734(0.7696)	−0.5793(0.7504)	13.706∗∗∗(0.5301)	−0.4133(0.7498)	−0.2378(0.7925)	−0.1156(0.6597)
Edu_hh	−0.0113(0.0533)	0.0287(0.0503)	0.0267(0.0495)	0.0779(0.0493)	0.0645(0.0519)	0.0555(0.0489)
Agehh	−0.0091(0.0199)	−0.0362(0.0225)	−0.0064(0.0174)	−0.0025(0.0179)	0.0093(0.0174)	0.0189(0.0176)
FS_hh	0.1518(0.1474)	0.2806∗∗(0.1459)	−0.1180(0.1384)	0.1848(0.1273)	0.0857(0.1312)	0.1469(0.1284)
TLUowned	−0.0285(0.0918)	0.0632(0.1156)	−0.0615(0.1042)	0.2334∗∗∗(0.0867)	0.2407∗∗(0.1029)	0.4188∗∗∗(0.0873)
Oxenowned	0.7242∗∗∗(0.2710)	0.8816∗∗∗(0.2785)	0.3055(0.3478)	0.6402∗∗(0.2651)	0.6939∗∗(0.3227)	0.9132∗∗∗(0.2694)
Off-farm	1.80e-06(0.00001)	0.000016(0.00001)	−0.00001(0.00001)	0.00002(0.00001)	2.09e-06(0.00001)	0.00001(0.00001)
Acc2crdt	0.0239(0.3888)	0.6835∗∗(0.3737)	−0.0289(0.3714)	0.2772(0.3631)	0.7931∗∗(0.3926)	0.7815∗∗(0.3488)
ExtCnt	0.2923∗∗(0.1199)	0.3519∗∗∗(0.1186)	0.5500∗∗∗(0.1156)	0.3583∗∗∗(0.1171)	0.5912∗∗∗(0.1150)	0.2908∗∗(0.1152)
Memb2institu	1.4506∗∗∗(0 0.5064)	−0.0764(0.4637)	0.9157∗∗(0.5442)	1.8238∗∗∗(0.6290)	−0.5058(0.4992)	0.4505(0.4936)
Dist2Mrod	0.0042(0.0087)	0.0008(0.0090)	0.0067(0.0087)	0.0066(0.0088)	0.0103(0.0087)	0.0028(0.0084)
DMmrktC	−0.0035(0.0098)	0.0041(0.0099)	−0.0084(0.0101)	−0.0131(0.0102)	−0.0124(0.0105)	0.0045(0.0099)
D2FaTC	−0.0072(0.0143)	−0.0042(0.0141)	0.0010(0.0145)	−0.0305∗∗(0.0174)	−0.0304∗∗(0.0168)	−0.0294∗∗(0.0149)
P_Numb	0.9452∗∗(0.0190)	0.0938(0.5012)	0.6215(0.4905)	0.4580(0.4509)	0.1869(0.4931)	1.1074∗∗(0.4320)
Plotdist	−0.0617∗∗∗(0.0222)	−0.0303(0.0190)	−0.0395∗∗∗(0.0103)	−0.0799∗∗∗(0.0292)	−0.0769∗∗∗(0.0233)	−0.0624∗∗∗(0.0176)
Plotsize	−0.0202(0.6584)	0.7148(0.6946)	0.7745(0.6179)	0.2853(0.6527)	1.0273∗∗(0.5206)	1.1534∗∗(0.5152)
_cons	−3.3770∗∗(1.4765)	−2.1312(1.6121)	−15.747∗∗∗(1.2836)	−3.8049∗∗(1.5498)	−3.1742∗∗(1.4619)	−6.4457∗∗∗(1.4114)

Log likelihood = −789.64742 Wald chi2(96) = 2890.21		Prob > chi2 = 0.0000 Number of obs = 527				

Among plot characteristics, farm households with greater plot sizes were also found to adopt combinations of improved maize seed and row spacing and all technology packages. Plot size is another important aspect to be considered wealthy, and it is a source of honor in rural communities of Ethiopia. Typically, farmers with larger plot sizes purchase commercial inputs and better practices agricultural innovations than farmers with lesser land size. This result is consistent with the results reported by Refs. [41,60]. Similarly, maize plots owned by the farm households influenced the improved maize seeds and the full technology package adoption positively and significantly. A farmer owning many plots practices the agricultural innovations better than farmers’ with one plot. The possible reason is that owning many plots reduces the chances of crop failure concurrently during unexpected risk and uncertainty. This, in turn, increases the confidence of farmers to adopt agricultural innovations and make appropriate investments. The result is in line with the result reported by Ref. [6].

Access to credit is among the institutional factors that affected the adoption of chemical fertilizer only, a combination of maize seed and row spacing, and full technology positively and significantly. Access to credit helps farmers to solve cash constraints during peak maize production season by supporting farmers to finance basic production inputs and labor force. Thus, it enhances the likelihood of maize technology adoption. This finding harmonizes with past findings of [23,26]. Farm households with a relatively high frequency of extension contact did adopt maize seeds only, chemical fertilizer only, row spacing only, a combination of improved maize seed and chemical fertilizer (IS_1_F_1_R_0_), improved maize seed and row planting, and a full technology package. Extension agents are sources of important information and technical knowledge that is related to input availability and technology application. This finding is consistent with the previous results of [58,61]. Furthermore, membership in any institutions was related positively to the adoption of improved maize seeds only, chemical fertilizer only, and both improved seeds and fertilizer jointly. Membership in farmer-based organizations supposed to be good sources of relevant information and production inputs. Hence, membership in farmer-based organizations enhances the likelihood of agricultural innovations' adoption. This finding is consistent with results of [62]. In contrast, the farmers' training center (FTC) distance was negatively related with the adoption of maize seeds and chemical fertilizer, maize seeds and row planting, and all three technology packages. The FTC that is far from the residence of farmers could reduce maize producers’ desire to participate in pilot demonstrations and training. This finding is in agreement with the results reported by Refs. [41,63]. Similarly, distance to maize plots was negatively associated with the adoption of improved maize seed only, row planting only, improved maize seeds and chemical fertilizer, improved maize seeds and row planting, and full technology package. When the residence of the farm households are distant from the maize plots, farmers start to hesitate and pay less attention to that plot because of additional resource requirement. This finding is consistent with the results reported by Refs. [43,64]. Table 5 shows the determinants of the individual or combinations adoption of maize technologies.

#### 3.7.2 The impact of multiple maize technology adoption on technical, allocative, and economic efficiency, and the food consumption score

The impact of improved maize seed, chemical fertilizer, and row planting adoption on the production efficiency and food security was analyzed by the MESR model and presented in Table 6. The model results showed that the adoption of the maize technology packages individually or in combination provides greater technical, allocative, and economic efficiency and food consumption scores, on average, than nonadoption does. However, in most cases, adopting a combination of two or more maize technology packages provides more technical, allocative, and economic efficiency and food consumption scores than adopting them individually. To estimate the accurate average adoption impact for farmers that adopted, the production efficiency and food security of the farmers that adopted maize technologies are compared with the production efficiency and food security if the maize farmers had not adopted. Similarly, the production efficiency and food security of the sample farmers that did not adopt any of the maize technologies are compared with the production efficiency and food security if the farmers had adopted the technologies.

**Table 6:** MESR model results of the adoption impact on outcome variables.

Outcome variables	Adoption category and effect of the treatment		Stage of the decision		Average effect of the treatment (Standard error)
			To adopt	Not to adopt	
Teffi	IS_1_F_0_R_0_	ATT	0.6559	0.4305	0.2254(0.0108)∗∗∗
		ATU	0.7211	0.4454	0.2756(0.0127)∗∗∗
	IS_0_F_1_R_0_	ATT	0.6120	0.4335	0.1785(0.0173)∗∗∗
		ATU	0.5422	0.4454	0.0967(0.0094)∗∗∗
	IS_0_F_0_R_1_	ATT	0.6240	0.4232	0.2008(0.0148)∗∗∗
		ATU	0.5344	0.4454	0.0890(0.0404)∗∗
	IS_1_F_1_R_0_	ATT	0.7306	0.4488	0.2818(0.0097)∗∗∗
		ATU	0.6740	0.4454	0.2285(0.0073)∗∗∗
	IS_1_F_0_R_1_	ATT	0.6907	0.4361	0.2546(0.0130)∗∗∗
		ATU	0.6067	0.4454	0.1613(0.0071)∗∗∗
	IS_1_F_1_R_1_	ATT	0.8014	0.4694	0.3320(0.0079)∗∗∗
		ATU	0.8054	0.4454	0.3600(0.0048)∗∗∗
Aeffi	IS_1_F_0_R_0_	ATT	0.6727	0.4771	0.1956(0.0160)∗∗∗
		ATU	0.5992	0.4955	0.1037(0.0103)∗∗∗
	IS_0_F_1_R_0_	ATT	0.7413	0.4839	0.2575(0.0175)∗∗∗
		ATU	0.8117	0.4955	0.3161(0.0106)∗∗∗
	IS_0_F_0_R_1_	ATT	0.8088	0.4508	0.3579(0.0174)∗∗∗
		ATU	0.7418	0.4955	0.2463(0.0917)∗∗∗
	IS_1_F_1_R_0_	ATT	0.8770	0.5126	0.3643(0.0110)∗∗∗
		ATU	0.8295	0.4955	0.3339(0.0082)∗∗∗
	IS_1_F_0_R_1_	ATT	0.8317	0.4897	0.3419(0.0161)∗∗∗
		ATU	0.7496	0.4955	0.2540(0.0074)∗∗∗
	IS_1_F_1_R_1_	ATT	0.8986	0.5330	0.3656(0.0118)∗∗∗
		ATU	0.9015	0.4955	0.4060 (0.0060)∗∗∗
Eeffi	IS_1_F_0_R_0_	ATT	0.5876	0.3067	0.2808(0.0154)∗∗∗
		ATU	0.7709	0.3177	0.4531(0.0184)∗∗∗
	IS_0_F_1_R_0_	ATT	0.5954	0.3036	0.2918(0.0132)∗∗∗
		ATU	0.6535	0.3177	0.3358(0.0092)∗∗∗
	IS_0_F_0_R_1_	ATT	0.5284	0.3052	0.2232(0.0124)∗∗∗
		ATU	0.3794	0.3177	0.0616(0.0428)∗∗
	IS_1_F_1_R_0_	ATT	0.7280	0.3254	0.4026(0.0093)∗∗∗
		ATU	0.8193	0.3177	0.5015(0.0184)∗∗∗
	IS_1_F_0_R_1_	ATT	0.6988	0.2997	0.3990(0.0101)∗∗∗
		ATU	0.6517	0.3177	0.3339(0.0065) ∗∗∗
	IS_1_F_1_R_1_	ATT	0.7778	0.3022	0.4755(0.0071)∗∗∗
		ATU	0.7945	0.3177	0.4767(0.0062)∗∗∗
FCS	IS_1_F_0_R_0_	ATT	34.848	24.098	10.749(0.6986)∗∗∗
		ATU	38.123	23.877	14.245(1.2852)∗∗∗
	IS_0_F_1_R_0_	ATT	33.870	24.163	9.706(0.8755)∗∗∗
		ATU	33.790	23.877	9.706(0.8755)∗∗∗
	IS_0_F_0_R_1_	ATT	34.605	23.955	10.650(0.6497)∗∗∗
		ATU	31.279	23.877	7.4012(0.5909)∗∗∗
	IS_1_F_1_R_0_	ATT	38.076	23.681	14.394(0.5124)∗∗∗
		ATU	34.926	23.877	11.048(0.3564)∗∗∗
	IS_1_F_0_R_1_	ATT	33.344	23.931	9.4128(0.9372)∗∗∗
		ATU	39.374	23.877	15.496(1.3124)∗∗∗
	IS_1_F_1_R_1_	ATT	40.671	23.603	17.068(0.4033)∗∗∗
		ATU	31.195	23.877	7.3171(0.5593) ∗∗∗

Source: Model output. ∗∗∗ & ∗∗ represent 1 & 5 % significance levels, respectively. The subscripts represent_1_ = adoption and _0_ = non-adoption. IS=Improved maize seed, F = chemical fertilizer, R=Row planting

The average treatment effect on treated (ATT) results revealed that, in all cases, the farm households that adopted maize technology packages in combination realized greater technical efficiency (Teffi) than adopters of the technology packages separately. The simultaneous adoption of improved maize seed, chemical fertilizer, and row planting (IS_1_F_1_R_1_) increased the Teffi of the farm households by 33 %. Likewise, the Teffi of the farmers that did adopt both improved maize seed and chemical fertilizer (IS_1_F_1_R_0_) in combination increased by 28 % followed by the farm households whose Teffi increased by 25 % when they adopted improved seed and row planting jointly (IS_1_F_0_R_1_). Similarly, the results from the average treatment effect on untreated (ATU) demonstrates that farmers that did not adopt maize technologies would have achieved a Teffi increase of 36 % with full technology package adoption (IS_1_F_1_R_1_) concurrently, 27 % with the maize seed adoption in insolation (IS_1_F_0_R_0_), and 22 % with the joint adoption of maize seed and row planting. This is probably because farmers better combine scarce production resources and technologies optimally when they adopt technologies in combination rather than individually. This result is in line with results of [65,66], who suggested that improved technology adoption enhances production efficiency, and that adopters are more technically efficient than nonadopters are. In addition, the study by Ref. [65] has revealed that the adopters of improved maize varieties increased their production efficiency by 20–28 %. Another study by Ref. [66] conducted in Ghana also revealed that the technical efficiency of smallholder farmers has increased by 6%–8% by adopting improved maize varieties. Likewise, Ref. [67] has reported that farmers that adopted new maize varieties increased their technical efficiency by 4.2 % than farmers that did not adopt in Ethiopia. Ref. [68] estimated the technical efficiency of farmers that adopted improved maize varieties and the farmers that did not adopt in Abuja, Nigeria. The results revealed that farmers that adopted the varieties are 7 % more technically efficient than their counterparts. In comparison to these results, the findings of this study imply that adopting two or more maize technology packages in combination could provide more technical efficiency than adopting them in isolation.

With respect to allocative efficiency (Aeffi), farm households that adopted two or more maize technology packages attained greater Aeffi than adopters in isolation did (Table 6). The results from ATT demonstrate that the Aeffi of the farm households increased by 37 % when they adopted full packages (IS_1_F_1_R_1_) and Aeffi increased by 36 % when farm households adopted improved seed and fertilizer jointly. This was followed by a 35.8 % increase of the Aeffi by the adopters of row planting only. Likewise, the results from ATU suggest that the farmers that had not adopted maize technologies would have achieved a higher Aeffi if they had adopted them. The estimation results revealed that farmers would have achieved an increase of Aeffi of 31 % with the adoption of chemical fertilizer only, 33 % with the joint adoption of both maize seeds and chemical fertilizer, and 40 % with the adoption of the full package. Furthermore, the highest economic efficiency (Eeffi) was also realized when farmers adopted technology packages in combination rather than individually. The ATT indicates that 47 % increment of Eeffi were realized when the farm households adopted full packages simultaneously (Table 6). The second largest Eeffi, which was 40.25 %, was also achieved by the sample respondent that did adopt maize seeds and chemical fertilizer jointly, followed by the farmers that did adopt maize seed and row spacing in combinations, whose Eeffi increased by 39.9 %. Similarly, the results from ATU denote that the farmers that had not adopted the maize technologies would have achieved an Eeffi increase of 47.7 % with full package adoption, 50 % with the adoption of both improved maize seeds and chemical fertilizer, and 45 % with the adoption of improved seeds only. This result imply that farmers are more able to produce the observed output at minimum cost, given the input prices, when they adopt improved technology packages in combination than when they do so separately.

Like for the other outcome variables, the highest FCS was attained by farmers who adopted two or more technology packages than by those who adopted them in isolation. The ATT results revealed that the FCS of the adopters of full technology packages increased by 17. In addition, the FCS of the farm households that adopted improved seeds and fertilizer increased by 14.4, followed by those that adopted improved seeds only, whose FCS increased by 10.74 (Table 6). The results from ATU demonstrate that the sample respondent that had not adopted maize technologies would have achieved a higher FCS if they had adopted it. For example, farm households would have achieved FCS increase of 15.5 by adopting improved seed and row planting, 14.24 with adoption of improved seed only, and 11 by combining improved seed and fertilizer. The results indicate that the smallholder farmers could be more food secured by adopting any of the improved maize innovations. This finding is consistent with the results reported by Refs. [22,[54], [69],^70^].

The study by Ref. [22] has reported that the use of new maize varieties decreases the food insecurity problem in Ethiopia by 2.5 percent. Another study by Ref. [54] also reported that the adoption of improved wheat varieties increases food security in rural Tanzania. Ref. [70] has reported that the adoption of maize varieties improved the food security status of farm households in Ethiopia. In summary, these results suggest that the adoption of agricultural innovations significantly increases production efficiency and food security, and the utmost advantage is achieved when these technologies are adopted in combination rather than individually (Table 6).

## 4 Conclusion and recommendations

This study aimed to examine the impacts of multiple maize technology adoption on the technical, allocative, and economic efficiency and food consumption scores of farming households in selected districts of Sidama region of Ethiopia. The MESR model was employed to estimate the adoption impact on the production efficiency and food security of the farming households. The first stage of MESR model estimation revealed that male headed household, household size, land size, livestock unit, oxen size; several plots, extension contact, credit access, and involvement in farmers-based organizations were significant factors affecting the adoption decision in the study areas. Moreover, the second stage results of the MESR model revealed the adoption of the maize technologies individually or in combinations significantly increased smallholder farmers' technical, allocative, economic efficiency, and food consumption scores. However, farmers that did adopt two or more maize technologies simultaneously attained higher scores of technical, allocative, and economic efficiency, and food consumption scores than those who adopted these packages' adoption separately. Depending on the research result, the following points are forwarded for relevant policy directions and interventions as follows.

• The government should enhance the capacity of extension workers, inspire involvement in institutions, offer credit, supply factors of production at the right time for the right prices, and subsidize the production inputs significantly.
• The development partners intended to boost the livelihoods of the farming households via increasing agricultural growth should rely on the relevant findings identified by the study for important interventions.
• Agricultural policymakers aimed at enhancing the share of agriculture in the economic growth of the country and making the lives of people better off must take into account the key factors that may hinder or accelerate the uptake of the agricultural technologies, while formulating policies and approaches for agricultural transformation.
• The government and policymakers should be concerned about the negative environmental impact of increased use of chemical fertilizer on soil depletion and water contamination, and its contradiction with sustainable agricultural practices.
• Thus, the government, policymakers, and development partners should promote a mix of improved technology packages (e.g., chemical fertilizer) and sustainable agricultural practices (e.g., animal manure (compost) and crop rotation) to meet simultaneously the proposed food security and poverty reduction goals as well as to maintain sustainable agriculture.
• Agricultural research institutes and seed enterprises intended to produce seed varieties are expected to ensure compatibility, applicability, accessibility, profitability, and environmental safety while releasing improved seeds.
• Extension agents should discharge the responsibility of supporting farmers fully through technical knowledge of technology applications and providing relevant information.
• Farmers should combine two or more technology packages, strengthen social engagement, and upsurge involvement in farmers-based organizations, and see the development agents more frequently than before to obtain the basic technical knowledge and production inputs.

## CRediT authorship contribution statement

**Ashenafi Guye:** Writing – original draft, Visualization, Software, Project administration, Methodology, Investigation, Formal analysis, Data curation, Conceptualization. **Tewodros Tefera:** Writing – review & editing, Validation, Supervision, Methodology. **Million Sileshi:** Writing – review & editing, Validation, Supervision, Methodology. **Abdi-Khalil Edriss:** Writing – review & editing, Validation, Supervision, Methodology.

## Informed consent

All sample farmers were informed that consent to participate in the study and the informed consent form were loudly read to the sample respondents in the language that they can speak and listen clearly prior to the interview.

## Data availability statement

The authors confirm that the data will be made available upon request.

## Ethics approval

Review and/or approval by an ethics committee were not needed for this study because no human or animal tissues were involved in the investigations.

## Funding

This research work was financed by the Ethiopian Ministry of Education (MoE).

## Declaration of competing interest

The authors declare that they have no known competing financial interests or personal relationships that could have appeared to influence the work reported in this paper.

## Figures and Tables

**Table 7:** Simple falsification test of instrumental variables

Instrument variables	Model I(Mlogit)	Model II(OLS)			
	Adoption decision	Technical efficiency	Allocative efficiency	Economic efficiency	Food consumption score
	Coefficient (Standard error)	Coefficient (Standard error)	Coefficient (Standard error)	Coefficient (Standard error)	Coefficient (Standard error)
ExtCnt	0.5112 ∗∗∗ (0.1168)	−0.0021 (0.0045)	−0.0075 (0.0093)	−0.0045 (0.0033)	0.2755 (0.3266)
DMmrktC	−0.0145 ∗∗ (0.0076)	0.0001 (0.0004)	−0.0001 (0.0006)	−0.0001 (0.0002)	−0.0027 (0.0177)
D2FaTC	−0.0011 (0.0142)	−0.0002 (0.0006)	−0.0002 (0.0011)	0.0002 (0.0005)	−0.0384 (0.0434)
Plotdist	−0.0700 ∗∗∗ (0.0193)	0.0005 (0.0003)	0.0002 0.0006	0.0004 (0.0002)	0.0032 (0.0168)
_cons	−1.5277 ∗∗∗ (0.5588)	0.4392∗∗∗ (0.0287)	0.5056∗∗∗ 0.0439	0.3145∗∗∗ 0.0161	24.39∗∗∗ 1.1036
Log likelihood = −908.40341 Wald chi2(24) = 117.25 Prob > chi2 = 0.0000		F-test = 0.70 Prob > F = 0.5968	F-test = 0.26 Prob > F = 0.9058	F-test = 1.57 Prob > F = 0.1854	F-test = 0.55 Prob > F = 0.6992

∗∗, and ∗∗∗ denotes 5 and 1 % significance level.

**Table 8:** Endogeneity test (Hausman Test)

	Coefficient
$\chi^{2}$	0.000
Probability value	1.000

**Table 9:** estat vif

Explanatory variables	Variance inflation factor (VIF)	Tolerance(1/VIF)
Education of the household head	1.41	0.709691
Age of the household head	1.38	0.725239
Main market center distance	1.36	0.737632
Membership in institutions	1.53	0.654594
Oxen ownership	1.50	0.666444
Main road distance	1.30	0.771750
Extension services	1.25	0.801691
Family size	1.21	0.828954
Livestock ownership	1.20	0.832140
Plot size	1.12	0.895898
Plot number	1.11	0.897058
Off-farm activities	1.09	0.920611
Credit access	1.08	0.924201
Sex of the household head	1.07	0.937043
Plot distance	1.05	0.954350
Distance to farmers training center	1.05	0.955329

Mean VIF	1.23	

## Footnotes

1 The third-level administrative division in Ethiopia, which is managed by the local government, contains many kebele within it.

2 Lowest level of administration in Ethiopia.

3 Probability proportional to the size of the population.

4 F^1-4^ represents F statistics for technical, allocative, and economic efficiency and food consumption scores.

5 1$ = 56.704 ETB (NBE, April 12, 2024).
